# Effect of pregnancy on the uterine vasoconstrictor response to exercise in rats

**DOI:** 10.14814/phy2.12337

**Published:** 2015-03-24

**Authors:** Christopher J Lashley, David A Supik, James T Atkinson, Robert J Murphy, Kathleen P O'Hagan

**Affiliations:** 1 Program in Biomedical Sciences, College of Health Sciences, Midwestern UniversityDowners Grove, Illinois, USA; 2 Department of Physiology, Chicago College of Osteopathic Medicine, Midwestern UniversityDowners Grove, Illinois, USA

**Keywords:** Exercise, gestation, physical activity, pregnancy, rat, uterine blood flow

## Abstract

A major maternal adaptation in pregnancy is the large increase in uteroplacental blood flow that supplies the growing fetus with oxygen and nutrients. The impact of gestation on the dynamic uterine vasoconstrictor response to exercise in the rat, a common model for pathophysiological disorders in pregnancy remains unknown. We hypothesized that rats exhibit a robust uterine vasoconstrictor response to acute exercise that is attenuated in late pregnancy. Pregnant (P, *N* = 12) and nonpregnant (NP, *N* = 8) rats were instrumented chronically with a ultrasonic transit-time flowprobe and carotid arterial catheter to directly measure uterine artery blood flow (UtBF) and blood pressure (BP), respectively, at day 20 of gestation for 5 min of treadmill exercise (7 m/min; 6% grade). Preexercise UtBF [P, 2.1 (SD1.6) vs. NP, 0.5 (SD0.3) mL/min *P* < 0.01) and uterine artery conductance (UtC) [P, 2.1(SD1.7) vs. NP, 0.4 (SD0.2) mL/min × mmHg^−1^ × 10^−2^, *P* < 0.01] were higher in pregnant rats, whereas preexercise BP was lower in the pregnant rats [P, 111 (SD13) vs. NP, 126 (SD13) mmHg, *P* = 0.02]. Preexercise heart rate was similar [P, 457 (SD30) vs. NP, 454 (SD42), *P* = 0.3]. Exercise initiated rapid and sustained decreases in UtBF [Δ−47% (SD12)] and UtC [Δ−49% (SD12)] that were attenuated in the pregnant rats [UtBF, Δ−25% (SD20) and UtC, Δ−30% (SD20), *P* = 0.02]. The BP and heart rate responses to exercise were unaffected in late pregnancy (interaction term, *P* = 0.3). In rats, dynamic exercise induces a uterine vasoconstrictor response that is blunted during late gestation, a response that we observed previously in pregnant rabbits.

## Introduction

A major maternal adaptation in normal pregnancy is the large increase in uteroplacental blood flow that serves to supply the growing fetus with oxygen and nutrients (Thaler et al. 1990; Konje et al. 2001). The positive relationship between uteroplacental blood flow and fetal growth and well-being (Clapp 2003; Lang et al. 2003) has stimulated interest in the control of the uterine circulation during acute stress, such as physical exercise.

Dynamic exercise in the nonpregnant state is typically accompanied by a decrease in blood flow to the splanchnic circulation, which is due primarily to vasoconstriction mediated by the adrenergic system (Rowell et al. 1984; Hales and Ludbrook 1988; Dowell and Kauer 1993; Rowell 1993). A limited number of studies have documented decreases in blood flow to the uterus during treadmill exercise in the nonpregnant rat using microsphere technology (Dowell and Kauer 1997) and in the nonpregnant rabbit using transit-time ultrasonic flowprobes (O'Hagan and Alberts 2003; Nesbitt et al. 2005). In rabbits, we found that the uterine vasoconstrictor response to treadmill exercise, as indicated by changes in uterine artery conductance, was unaffected in early pregnancy (−Δ45%) but greatly attenuated at mid-gestation and term gestation (−Δ2%) (Nesbitt et al. 2005). These studies in the rabbit suggest that normal circulatory adaptations at term pregnancy limit the expected redistribution of blood flow away from the uteroplacental circulation during short-term exercise stress.

There is evidence that uterine circulatory control mechanisms are impacted in diabetes and hypertensive disorders of pregnancy (Hackett et al. 1992; Knock et al. 1997; Pietryga et al. 2005). The rodent is a common model used to study pathophysiologic consequences of diabetes and pregnancy-induced hypertension on the uteroplacental and systemic circulations (Chartrel et al. 1990; Stanley et al. 2009; Li et al. 2012; Gokina et al. 2013). There is increased interest in the potential of chronic exercise to affect maternal and postnatal consequences of hypertensive disorders or diabetes during pregnancy (Genest et al. 2012; Gilbert et al. 2012; Damasceno et al. 2013). Thus, in the rat, an understanding of the impact of pregnancy on uterine artery hemodynamic responses to acute exercise stress is desirable. In this study, we hypothesized that rats, similar to rabbits, exhibit a robust uterine vasoconstrictor response to short-term treadmill exercise that is attenuated in late pregnancy. We tested this hypothesis by measuring uterine artery blood flow in nonpregnant and late pregnant rats for 5 min of treadmill exercise.

## Methods

### Animal care and handling

All animal care and experimental procedures were approved by the Midwestern University IACUC and performed on Midwestern University Downers Grove campus.

Female Sprague–Dawley rats weighing 100–124 g (22–29 days) were obtained from an approved vendor (Harlan Laboratories, Indianapolis, IN and Charles River Laboratories International, Wilmington, MA). Rats were placed on a 12-h light/12-h dark cycle with lights on at 6AM. Rats were handled for approximately 5 min 3–5 days/week to become accustomed to handling during trials. Once instrumented or bred (pregnant), all animals were individually housed in cages. Complete sets of usable data were obtained from 20 animals, 12 in the pregnant group and 8 in the nonpregnant group.

### Treadmill familiarization

Rats were familiarized with treadmill running 1–2 × per week for at least 4 weeks prior to surgery or breeding. As part of the familiarization process, the rat was allowed to explore the treadmill area and then exercised as follows: 5 min @ 7 m/min at 6% grade, 13 m/min at 6% grade, and 20 m/min at 6% grade with at least 3 min separating the bouts of exercise.

### Breeding

Sprague–Dawley female rats are able to breed at 60–70 days of age. For estrous cycle staging, vaginal secretions were collected daily (8 am–12 pm) using a clean pipette filled with 15 *μ*L of normal saline and examined using light microscopy. When epithelial cell morphology indicated proestrous or estrous, the female rat was placed in the male rat's cage between 1 and 3 pm, left overnight and returned to the home cage in the morning (Marcondes et al. 2002). If a sperm plug was not observed, a vaginal wash (see above) was performed to assess the presence of sperm. Day 0 of gestation was assigned at this time. If there was no evidence of copulation, the estrous cycle staging and breeding was repeated. Estrous cycle staging was not performed in the rats in the nonpregnant group.

### Surgical preparation

The rats underwent sterile surgery to implant a flowprobe on the right uterine artery for the measurement of uterine artery blood flow (UtBF) and a polyurethane catheter in the left carotid artery for measurement of blood pressure (BP). In the pregnant group, the surgery was performed on day 10–14 of gestation. Animals were anesthetized using an intraperitoneal injection of a ketamine (80 mg/kg)/xylazine (2.5 mg/kg) mixture, and the surgical sites were prepared for surgery. Prior to surgery, ketoprofen was administered (3.0 mg/kg s.c.) for analgesia. Surgical anesthesia was maintained with 1.0–1.5% isoflurane in 100% O_2_ gas using a rodent mask and Bain anesthesia circuit. Ketoprofen administration was repeated 8–12 h after the initial analgesic dose and on the first postoperative day.

#### Implant uterine artery flowprobe

Transonic Systems maintains on their website (www.transonic.com) detailed illustrated technical bulletins that assist the surgeon with chronic implantation of vascular probes in research applications. The following is our adaptation of these procedures to the rat uterine artery. We emphasize that there are alternative ways to effectively approach each of the steps described below.

After routine skin preparation for surgery, a midline abdominal skin incision and small midscapular skin incision were made. A Transonic Systems™ (Ithaca, NY) ultrasonic transit time flowprobe (0.5–0.7 mm PSB) was routed subcutaneously from the midscapular incision to the lower abdomen. The lower abdominal cavity was then opened along the midline and the abdominal walls retracted, which provided visual access to the cervical end of the bicornate uterus. A small segment of the right uterine artery running parallel to the cervical end of the uterine horn was isolated from surrounding adipose tissue and the uterine vein using Dumont™ 5/45 INOX forceps (tip 0.05 × 0.01 mm; Roboz Surgical Instruments, Gaithersburg MD). In pregnant animals, this site basically corresponded to the arterial segment between the first (cervical) and second fetal site. This area is readily accessible to the surgeon without disturbing the rest of the pregnant uterine horn. A small rectangular (approximately 4 × 1 cm) piece of sterile polyester surgical mesh (Mersilene; Ethicon, Somerville, NJ) was trimmed at midpoint (to approximately 3–4 mm) in the surgeon's estimation to allow the mesh to lie flat under the artery without impinging on adjacent arcuate arteries branching off the main uterine trunk. After the mesh was placed under the artery, the probe was positioned around the artery. The two ends of the mesh rectangle were brought up and together at the probe lead. The complex was secured with a silk suture placed through the mesh ends and tied around the probe lead. At this point, the probe-mesh complex is allowed to tilt toward and perhaps rest gently on the uterine horn (as the lead exits through the midline incision) and care is taken that the probe is not pulling upward on the artery. A small amount of surgical lubricant was placed in the probe window (to prevent invasion of the liquid Kwik Cast elastomer) and a small piece of sterile polyester felt was then placed on each side of the probe-mesh complex. A rapid-cure silicone elastomer (Kwik Cast; WPI Instruments, Sarasota, FL) was applied to stabilize the probe and mesh complex. The abdominal cavity was flooded with warm normal saline and the abdominal cavity was closed using absorbable suture. After moving the rat to the prone position, the externalized end of the probe lead was passed through a small piece of polyester surgical mesh that was placed in the subdermal space of the midscapular skin incision. The exterior probe lead connector was stabilized in a rigid cuff (Transonic Systems) that was secured to the skin, subcutaneous polyester surgical mesh and underlying musculature with nylon sutures.

#### Implant chronic carotid artery catheter

A polyurethane catheter (3Fr; Instech, Plymouth Meeting, PA) was placed in the left common carotid artery. The catheter was exteriorized to the back, just rostral to the midscapular incision and connected to a miniature vascular access port that was secured in a lightweight harness worn by the rat (Instech). The catheter was filled with a lock solution of 0.3 mL heparinized saline (500 IU/mL) that was replaced daily.

### Experimental protocol

The chronically instrumented rats were brought to the laboratory for the treadmill experiment at day 20 of gestation for the pregnant animals or at 6–9 days post surgery for the nonpregnant animals. Pulsatile UtBF and BP were monitored continuously using a computer based data acquisition system (Powerlab 8SP; LabChart 5.0 ADInstruments, Colorado Springs, CO). Heart rate (HR) was derived from the BP or UtBF pulsatile signal. Uterine artery conductance (UtC) was calculated as UtBF/BP.

The rat was placed on the treadmill unrestrained. After 30–45 min, at least 2 min of hemodynamic data were collected during a nonactive period immediately prior to the initiation of exercise (Preexercise). Data were collected continuously for a 5-min exercise bout (Exercise) at 7 m/min, 6% grade. The lowest of the three treadmill familiarization workloads was selected for the experiment because the pregnant rats were compliant with this workload for the 5-min exercise period. If necessary, rats were encouraged to run by lightly tapping on the tail. Following exercise, data were continuously recorded for two additional minutes (Recovery).

### Euthanasia

Rats were euthanized with CO_2_ overdose, followed by pneumothorax in dams and decapitation of fetuses. Pregnant rats were euthanized on day 21 of gestation. Fetal number and weight in each uterine horn were recorded.

### Statistical analysis

A two-way ANOVA with repeated measures utilizing a general linear model (PASW18, Chicago, IL) was performed to compare the effect of pregnancy status (main effect of group; nonpregnant, pregnant) on the absolute physiological responses to exercise or recovery from exercise (main effect of time). In Figure2, the first minute of exercise or recovery is plotted in consecutive 10 s intervals to illustrate the transitional responses. In the statistical analyses, the data for the consecutive 5 min of exercise and 2 min of recovery were represented by 60 s averages for each minute. The main effect of time and the group by time interaction term produced by the ANOVA were explored using planned simple linear contrasts. Simple linear contrasts compared the preexercise value to subsequent time intervals.

A two-way ANOVA utilizing a general linear model was performed to compare the relative (%) UtC and UtBF responses to exercise or recovery between groups (nonpregnant, pregnant) with repeated measures on the main effect of time. The group by time interaction term for the exercise ANOVA (5 levels) was explored using planned simple linear contrasts. Simple linear contrasts compared the first minute of exercise to subsequent time intervals. Contrast analyses were unnecessary for the recovery ANOVA as the main effect of time had only two levels. In Figure3, the first minute of exercise or recovery is plotted in consecutive 10 s intervals to illustrate the transitional responses. In the statistical analyses, the data for the consecutive 5 min of exercise and 2 min of recovery were represented by 60 s averages for each minute.

Values without a time component were compared using paired or unpaired Student's *t*-test, as appropriate. Statistical significance was set at *P* < 0.05. Values are stated as mean (standard deviation; SD) (Curran-Everett and Benos 2004).

## Results

### Body weight and fetal outcomes

The pregnant animals were heavier than the nonpregnant rats at the time of the experiment [320 g (SD35) vs. 273 g (SD47), *P* = 0.02]. Table1 shows the fetal outcomes for the pregnant (*N* = 12) and the nonpregnant groups (*N* = 8). There was no difference in total viable pup number, weight per individual fetus, and total fetal weight in the probe-instrumented uterine horn versus the contralateral noninstrumented horn. At the time of euthanasia, there were three fetuses (from two dams) in the noninstrumented horn that were judged nonviable prior to euthanasia based on small size (1.6–2.5 g) and dark coloration. Retrospectively, we estimated the number of resorptions in each horn for each dam using casual observations recorded at the time of euthanization and the difference in estimated fetal count at surgery versus the fetal count at the time of euthanization. It appears that eight of the 12 pregnant rats experienced a resorption event (median = 1, range 0–4) in the instrumented horn and three of 11 pregnant rats with fetuses in the noninstrumented horn experienced a resorption event (median = 0, range 0–2). Three pregnant rats experienced no obvious resorptions in either horn. In most cases, the resorption events in the instrumented horn occurred at the cervical end of the horn, in close proximity to the probe implantation site. We infer that the resorptions occurred early in the recovery from surgery. The greater proportion of resorptions in the instrumented horn is likely related to the more extensive physical handling of the uterus and local vasculature associated with implantation of the probe.

**Table 1:** Fetal number and weight

	Uterine horn instrumented	Uterine horn noninstrumented	Paired *t*-test (2-tailed) *P* value
Mean fetal number	4 (SD2)	5 (SD4)	0.22
Mean fetal weight in horn (g)	16 (SD9)	19 (SD13)	0.34
Mean weight per fetus (g)^1^	4.2 (SD1.2)	4.1 (SD1.1)	0.68

1 *N* = 11; Data from one pregnant rat was dropped from the paired *t*-test as there was no fetus carried in the noninstrumented horn.

**Figure 1:**
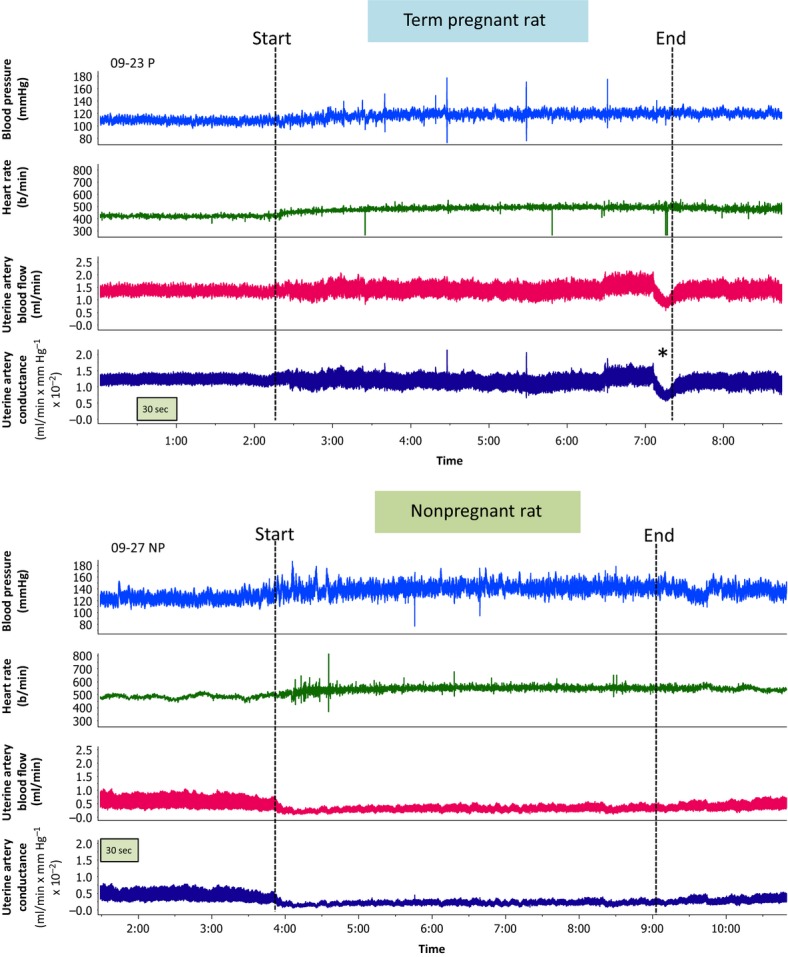
Screen shots of original data tracings of a pregnant (top) and a nonpregnant rat (bottom) during an exercise trial at 7 m/min at 6% grade. In this image, the signal marker channel was replaced by two vertical dashed lines indicating the start and stop of the treadmill belt, and the channel labels were replaced for clarity. The tracing from the nonpregnant rat best illustrates the rapid decrease in uterine blood flow and conductance at the onset of exercise and the rapid return toward resting values at the cessation of exercise. The exercise vasoconstrictor response is greatly attenuated in the pregnant rat. The transient vasoconstriction marked by the asterisk (*) observed in the pregnant rat at the end of the exercise period occurred when the investigator touched the rat's tail to encourage running effort. This rapid response suggests that neural modulation of the uterine vasculature is possible in this pregnant rat, despite the greatly attenuated vasoconstrictor response to dynamic exercise.

Figure1 is composed of screen shots from the data acquisition software that present the hemodynamic responses before, during and after treadmill exercise in a term pregnant (top tracing) and a nonpregnant rat (bottom tracing). The onset of exercise in the nonpregnant rat was associated with an abrupt decrease in UtBF and UtC that persisted during the 5 min of exercise and largely reversed during the 2-min recovery period. The tracing from this particular pregnant rat illustrates a substantial attenuation of the uterine artery vasoconstrictor response to treadmill exercise, as there was little change in UtC during the exercise period. The transient reduction in UtBF and UtC observed in the pregnant rat at the end of the exercise period (noted with an asterisk) occurred when the investigator touched the rat's tail to encourage running effort. This rapid response suggests that neural modulation of the uterine vasculature was not abrogated to all stimuli in this pregnant rat, despite the greatly attenuated vasoconstrictor response to dynamic exercise.

### Heart rate and blood pressure responses

During the preexercise period (on treadmill, but not moving about), HR (b/min) was similar (*P* = 0.85) in the nonpregnant group [454 (SD42)] and the pregnant group [457 (SD30)]. HR increased (main effect of time, *P* < 0.001) during exercise and was similar over minutes 2–5 of exercise [Nonpregnant: 512 (SD32) vs. Pregnant: 496 (SD27), *P* = 0.3]. HR remained elevated in the 2nd minute of recovery (main effect of time, *P* < 0.001) with no difference in response pattern between the groups (interaction term; *P* = 0.3).

**Figure 2:**
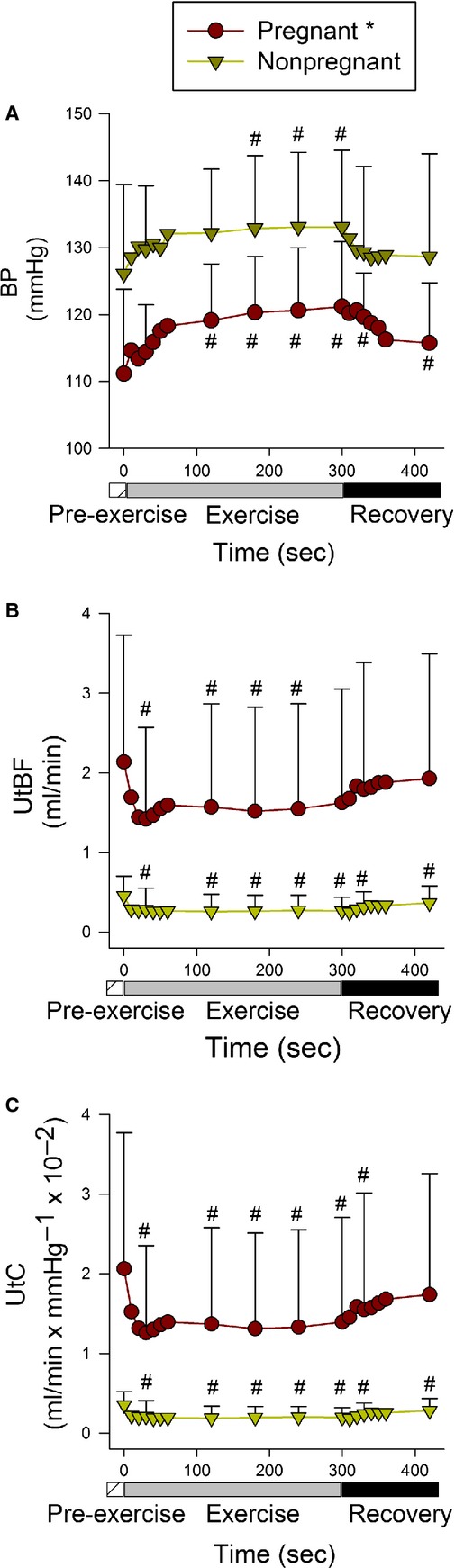
Mean arterial blood pressure (BP, panel A), uterine artery blood flow (UtBF, panel B), and uterine artery conductance (UtC, panel C) for the nonpregnant (*N* = 8) and pregnant (*N* = 12) rats at preexercise (2 min average), during 5 min of exercise at 7 m/min, 6% grade, and during 2-min recovery post exercise. The first minute of exercise and recovery are plotted in 10 s intervals to illustrate the dynamic responses at the beginning and end of exercise. Values are mean (SD). *P < 0.05 for BP, *P* < 0.01 for UtBF and UtC, Pregnant versus Nonpregnant (main effect of group). ^#^*P* < 0.05 Exercise or Recovery one minute average versus preexercise 2 min average, within group comparison. ANOVA indicated no significant group × time interaction for exercise or recovery for BP, UtBF or UtC.

Figure2A depicts mean BP for the nonpregnant and pregnant groups during preexercise, 5 min of exercise at 7 m/min, 6% grade and 2 min of recovery. At preexercise, BP (mmHg) in the pregnant rats [111(SD13)] was lower (*P* = 0.02) than BP in the nonpregnant rats [126 (SD13)]. BP increased over duration of the exercise (Time, *P* < 0.01) and pregnancy did not affect the pattern of the BP response to exercise (interaction term, *P* = 0.6). During steady-state exercise (averaged over minutes 2–5), BP was lower in the pregnant rats [Pregnant: 120 (SD9) vs. Nonpregnant: 131 (SD10), *P* = 0.02]. As illustrated in Figure2A, BP rapidly recovered toward preexercise values during the 2-min postexercise period (main effect of time, *P* = 0.01) and pregnancy did not affect the recovery response (interaction term, *P* = 0.3).

### Uterine artery blood flow and conductance responses

Figure2B shows the absolute UtBF responses for the nonpregnant and pregnant rats. UtBF (mL/min) was elevated in the pregnant animals [Nonpregnant: 0.46 (SD0.25) vs. Pregnant: 2.14 (SD1.59); *P* < 0.01) and remained so during steady-state exercise [average of min 2–5; Nonpregnant: 0.26 (SD0.19) vs. Pregnant: 1.57 (SD1.33), *P* < 0.01] and through recovery. Averaged over groups, UtBF decreased rapidly at the onset of exercise and remained lower than the preexercise level through the 5 min of exercise (main effect of time, *P* = 0.02; Rest vs. min 5, *P* = 0.03). The UtBF response to exercise was not affected by pregnancy status (interaction term, *P* = 0.2). As illustrated in Figure2B, UtBF rapidly returned toward the preexercise level at the cessation of exercise (main effect of time, *P* = 0.06). Pregnancy did not affect the recovery response (interaction term, *P* = 0.6).

**Figure 3:**
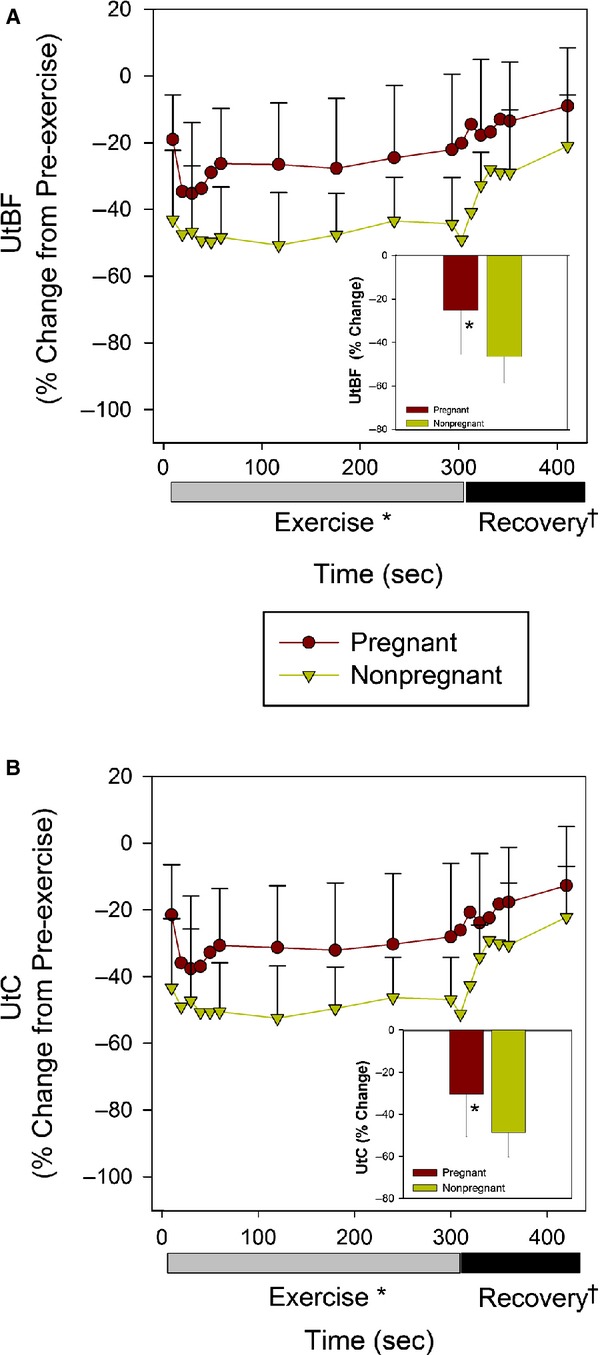
Percent change in UtBF (A) and percent change in UtC (B) from the preexercise average for the nonpregnant (*N* = 8) and pregnant (*N* = 12) groups during 5 min of exercise at 7 m/min 6% grade, and during recovery post exercise. The inset graphs depict the steady-state responses to exercise (average of minutes 2–5) in each group. The first minute of exercise and recovery are plotted in 10 s intervals to illustrate the dynamic responses at the beginning and end of exercise. Values are mean (SD). *P < 0.05 Pregnant versus Nonpregnant (main effect of group). ^†^*P* < 0.01 Recovery minute 1 versus minute 2. ANOVA indicated no group × time interaction during exercise or recovery for the percent change in UtBF or UtC.

The dynamic responses of UtBF to exercise are illustrated in Figure3A, which depicts the percent change in UtBF from the preexercise period for the nonpregnant and pregnant groups during exercise and recovery. The relative decrease in UtBF at steady-state exercise (average of minutes 2–5) in the pregnant group was 53% of the UtBF response in the nonpregnant group [Nonpregnant: Δ−47% (SD12, Δ−36 to −Δ57% [95%CI]) vs. Pregnant: Δ−25% (SD20, Δ−12 to −Δ38% [95%CI]), *P* = 0.02]. There was no significant interaction (*P* = 0.6) between gestational state and exercise, indicating that the dynamics of the UtBF response during minutes 1–5 of exercise were unaffected by pregnancy, although significantly different in magnitude (main effect of group, *P* = 0.02). The dynamics of the UtBF response during the 2-min postexercise period (main effect of time, *P* < 0.01) was unaffected by pregnancy (interaction term, *P* = 0.2).

As observed for UtBF, UtC (Fig.2C, mL/min × mm Hg^−1^ × 10^−2^) was significantly elevated in the pregnant animals during the preexercise period [Nonpregnant: 0.35 (SD0.17) vs. Pregnant: 2.06 (SD1.71), *P* < 0.01] and remained so during the exercise and recovery periods. Averaged over groups, UtC abruptly decreased at the onset of exercise and remained lower over the duration of the 5 min exercise (main effect of time, *P* < 0.01; Rest vs. min 5, *P* = 0.01). The UtC response to exercise was not affected by pregnancy status (interaction term, *P* = 0.08). As illustrated in Figure2C, the vasoconstrictor response returned toward preexercise levels at the cessation of exercise (main effect of time, *P* < 0.05). Averaged across groups, UtC was at near preexercising levels during the 2nd minute of recovery (Rest vs. min 2, *P* = 0.05). The recovery of UtC postexercise was not affected by pregnancy (interaction term, *P* = 0.2).

Relative changes in vascular conductance reflect relative changes in vessel vasoconstriction in the exercise situation where flow to a vascular bed changes more than mean arterial pressure (O'Leary 1991; Buckwalter and Clifford 2001). The relative decrease in UtC in the pregnant rats during steady-state exercise (Fig.3B inset) was attenuated compared to the nonpregnant group [Nonpregnant: Δ−49% (SD12, Δ−39 to Δ−58% [95%CI])]; Pregnant: Δ−30% (SD20, Δ−18 to Δ−43% [95%CI]), *P* = 0.02]. As illustrated in Figure3B, there was no interaction between gestational state and time with exercise (*P* = 0.6) indicating that the magnitude (main effect of group, *P* = 0.03) but not the pattern of the vasoconstrictor response during treadmill exercise were affected at term pregnancy in the rat. Postexercise, UtC recovered toward the preexercise level (main effect of time, *P* < 0.01) and pregnancy did not affect this response (interaction term, *P* = 0.3).

## Discussion

In this study, we used ultrasonic transit-time technology to measure volume flow in a uterine artery in pregnant and nonpregnant rats. To our knowledge, this is the first time dynamic volume flow measurements in the uterine circulation have been made during exercise in rodents.

The major findings in this study were that (1) a brief bout of treadmill exercise induced a sustained reduction of approximately 50% in uterine artery blood flow and conductance in nonpregnant female rats, and (2) while a reduction in uterine artery conductance during exercise was also observed in term pregnant rats, the magnitude of the vasoconstrictor response was attenuated by approximately 39%. These results are qualitatively similar to our findings in rabbits (O'Hagan and Alberts 2003; Nesbitt et al. 2005) and lend support to the hypothesis that advancing gestation is associated with a reduction in exercise-induced vasoconstriction in the uterine circulation.

Surprisingly, there is less known of the uterine artery blood flow response to dynamic exercise in the nonpregnant state than in the pregnant state. Most studies targeting uterine blood flow and exercise in women and animals utilize only pregnant subjects, which precludes analysis of physiologic adaptations in dynamic vascular control in this circulation. Dowell and Kauer (Dowell and Kauer 1993) utilized radioactive microspheres to quantitate uterine blood flow at rest and during steady-state treadmill exercise in nonpregnant rats. At a workload that apparently elicited minimal changes in HR or BP, they found no change in total uterine flow accompanied by a 24% increase in calculated uterine vascular resistance. However, our dynamic measurements of uterine artery blood flow in the nonpregnant rat at a moderate workload that elicited increases in HR and BP (Figs.1, 2) clearly illustrate that a decrease in uterine artery flow and conductance occurs in the first 10 s of treadmill exercise and peaks within 30–40 s of the onset of exercise. The uterine vasoconstriction (∽Δ−50%) was well-maintained over the 5 min of exercise. In previous studies from our laboratory, a similar uterine vasoconstrictor response was observed in nonpregnant rabbits undergoing moderate to maximal treadmill exercise (O'Hagan and Alberts 2003; Nesbitt et al. 2005). As with the mesenteric and renal circulations (Hales and Ludbrook 1988; Mueller et al. 1998), it appears the uterine circulation participates in the redistribution of cardiac output during moderate to heavy dynamic exercise. The rat uterine vasculature is innervated by sympathetic nerves (Haase et al. 1997; Klukovits et al. 2002; Gnanamanickam and Llewellyn-Smith 2011) and electrical activation of sympathetic nerves results in uterine vasoconstriction that is blunted in the presence of alpha-adrenergic receptor antagonism (Fuller et al. 1979; Hutchison et al. 1997). Thus, it is likely that the rapid onset of uterine vasoconstriction at the beginning of exercise is primarily driven by activation of uterine sympathetic nerves.

A major finding in our study is that the exercise-initiated uterine vasoconstrictor response in rats is blunted at term pregnancy, a finding we had previously observed in rabbits (O'Hagan and Alberts 2003; Nesbitt et al. 2005). The gestational attenuation of exercise uterine artery vasoconstriction in rabbits is expressed in the last third of gestation (Nesbitt et al. 2005). It is probable, but unproven, that the gestational attenuation of exercise uterine vasoconstriction in rats, similar to rabbits, is only present during late gestation. In term pregnant rabbits (day 28 of a 30–31 day gestation) exercised to volitional fatigue (Nesbitt et al. 2005), the average change in uterine artery conductance was −2% (SD24). In contrast, the average change in uterine artery conductance was −30%(SD20) in the term pregnant rats in this study (day 20 of a 21–22 day gestation) at an exercise intensity that elicited approximately 75–90% of estimated maximal HR [600 bpm, (Gonzalez et al. 1993)]. It may be that the vasoconstrictor response to exercise at term gestation is more pronounced in the pregnant rat than in the rabbit. An additional factor to consider when making quantitative comparisons between the rat and rabbit, in addition to the difference in the exercise stimulus, is the timing of the experimental measurements in the rat. Dowell and Kauer (Dowell and Kauer 1997) found that fetal weight increased threefold and total uterine blood flow, as measured with the radioactive microsphere technique, more than doubled between day 19 and day 22 of rat gestation. Markers of cell division rates in the main uterine artery are still elevated at day 20 in rat pregnancy (Cipolla and Osol 1994), indicating active arterial remodeling continues very late into rat pregnancy (Osol and Moore 2014). More broadly, this suggests that structural or functional gestational adaptations that lead to attenuation of the exercise vasoconstrictor response for a given rat are still developing between 19 and 22 days of gestation, which means our measurements at day 20 may not reflect the point of peak attenuation for every rat.

Uteroplacental blood flow decreases to varying degrees during moderate to heavy treadmill exercise in sheep [−13%, (Lotgering et al. 1983)], goats [−18%, (Hohimer et al. 1984)], and rabbits [−16% (O'Hagan and Alberts 2003) to +7% (Nesbitt et al. 2005)]. In this study, uterine artery blood flow in the day 20 pregnant rat decreased by 25%. Quantitatively, this finding compares favorably with the estimated (from Fig.1) relative decreases in total uterine blood flow observed by Dowell and Kauer (Dowell and Kauer 1993) in the day 19 (−15%) and day 22 (−25%) pregnant rat exercised using an submaximal exercise intensity (8.5 m/min, 0% grade). In contrast, Jones et al. (Jones et al. 1990) observed a 72% to 65% decrease in blood flow (microsphere technique) to the uterus and placenta, respectively, in day 17 pregnant rats treadmill-exercised at 30 m/min. It may be that this extreme reduction in uterine blood flow, which exceeds the mean responses in nonpregnant rabbits (O'Hagan and Alberts 2003; Nesbitt et al. 2005) and nonpregnant rats (Dowell and Kauer 1993), was influenced by a combination of the mid-gestation timing of the study and the high intensity of the exercise.

Mechanisms underlying the gestational attenuation of uterine artery vasoconstriction during exercise are not clear but may involve a combination of changes to central and peripheral vascular control mechanisms associated with the normal adaptive responses to pregnancy. In the rat, late pregnancy is characterized by a reduction in the density of adrenergic nerves (Brauer and Smith 2015) associated with the smaller myometrial blood vessels, as detected by tyrosine hydroxylase-immunohistochemistry (Haase et al. 1997; Gnanamanickam and Llewellyn-Smith 2011) or glyoxylic acid fluorescence histochemistry (Klukovits et al. 2002). In addition, it is likely that the larger extrauterine vessels retain a higher degree of adrenergic innervation than the much smaller myometrial vessels, as has been observed in the main uterine artery of the guinea pig (Mione and Gabella 1991) and the extrauterine (mesometrial) vasculature in the rat (Haase et al. 1997).

The uterine vasculature retains alpha-adrenergic responsiveness during late pregnancy, as indicated by a robust uterine circulatory vasoconstrictor response to intravenous phenylephrine in conscious term pregnancy rabbits (Nesbitt et al. 2005) and increased sensitivity to phenylephrine in the extrauterine radial arteries, a major site of uterine vascular resistance in the late pregnant rat (D'Angelo and Osol 1993; Osol and Cipolla 1993). However, it is not known if adrenergic receptor function is antagonized or opposed in the uterine blood vessels differentially during exercise due to upregulation of vasodilator systems in pregnancy, such as the endothelial-based nitric oxide (NO) system [for review, (Valdes et al. 2009)]. In isolated vessels, inhibition of NO synthase enhances alpha-adrenergic induced vasoconstriction in the main uterine artery (Ni et al. 1997; Xiao et al. 1999) which suggests that the NO system could oppose acute adrenergic-induced vasoconstriction in the conduit vessel.

Although there is evidence that sympathetic innervation of the uterine circulation is reduced at late pregnancy, functional sympathetic control of the uterine vasculature is still present. Acute activation of the nasopharyngeal reflex, which is known to strongly stimulate renal sympathetic nerve activity (Dorward et al. 1985; O'Hagan and Casey 1998), resulted in rapid and profound reductions in uterine artery blood flow and conductance that were similar in late pregnant and nonpregnant conscious rabbits (O'Hagan and Alberts 2003). It is likely the hemodynamic response to activation of the nasopharyngeal reflex is mediated by activation of uterine sympathetic nerves. Exercise in the rabbit, however, does not provoke maximal sympathetic outflow to viscera, as indicated by a renal sympathetic nerve activity response during exercise that was approximately 30% of nasopharyngeal reflex response at rest (O'Hagan and Alberts 2003). It is possible that relative uterine vasoconstrictor responses are blunted only at lower intensities of sympathetic neural stimulation, as observed in earlier studies in the anesthetized pregnant dog (Ryan et al. 1974) and sheep (Fuller et al. 1979).

In the course of our studies with the exercising pregnant rat, we have observed rapid and transient reductions in uterine blood flow superimposed on the steady-state exercise response when the animal is startled, as shown in the original data tracing (Fig.1). This observation reinforces the argument that in the pregnant rat, sympathetic outflow is not maximal during exercise and reserve activity can be reflexively recruited. More broadly, it leads to the question of whether gestational alterations in central sympathetic control during exercise also contribute to the blunted uterine vasoconstrictor response. It has been well-documented that late pregnancy in the rabbit and rat is associated with blunted arterial baroreflex control of HR and renal sympathetic nerve activity, and that this is primarily a centrally mediated phenomenon (Heesch and Rogers 1995; Brooks et al. 1997, 2012; O'Hagan et al. 2001). Definitive evaluation of this question requires direct measurements of uterine sympathetic nerve activity during exercise, which has not been reported. We do know that in the rabbit, the sympathetic outflow to the kidney during treadmill exercise is not altered during late gestation (O'Hagan and Alberts 2003).

Pregnancy in women is also associated with progressive increases in uterine artery flow and decreases in uterine vascular resistance in the resting state (Thaler et al. 1990). The resistance index (ratio of peak systolic to minimum diastolic waveforms) and pulsatility index (systolic-diastolic/mean waveform height) are used clinically as markers of uteroplacental circulatory resistance. Studies utilizing Doppler ultrasonography to measure uterine artery flow velocity waveforms in pregnant women undergoing submaximal upright cycle exercise in the third trimester report approximately 35% increases in the resistance index within 2 min postexercise (Morrow et al. 1989; Erkkola et al. 1992). Kennelly (Kennelly et al. 2003) reported a 20% increase in the right uterine artery pulsatility index 2 min after strenuous cycle exercise. As ultrasound imaging is restricted to end-exercise or pauses in the exercise protocol due to movement artifacts, it is likely that these studies somewhat underestimate the uterine vasoconstrictor response to exercise. Importantly, there have been no comparable measurements of the resistance or pulsatility indexes postexercise in nonpregnant or postpartum females, so it is still unknown if pregnancy in women is associated with an attenuated uterine vasoconstrictor response to exercise, as we have shown in rodent (this study) and rabbit (O'Hagan and Alberts 2003; Nesbitt et al. 2005) pregnancy.

### Limitations

As expected, the preexercise BP was lower in the term pregnant rats (Dowell and Kauer 1993, 1997; Brooks et al. 2010). The preexercise HR was similar (∽450 b/min) between the nonpregnant and pregnant rats. Pregnancy increases HR in rats at rest; for example, Brooks et al. (2010) observed that pregnancy resulted in an 20–25 b/min increase in average HR by Day 19 of gestation, whether measured remotely with telemetry over the 12 h light phase (∽400 b/min) or 12 h dark phase (∽455 b/min) while the animals were living in their home cages. A 25 beat differential between nonpregnant and Day 19 rats resting quietly in their cages was reported by Dowell and Kauer (Dowell and Kauer 1993). Although rats in our study were familiarized with the treadmill and the running protocol, it is likely the lack of differential in preexercise HR between groups reflects the interaction of pregnancy status, a heightened degree of arousal due to the treadmill environment and recent mild motor activity associated with being unrestrained on the treadmill. For example, Musch et al. (1998) measured HR in treadmill-familiarized nonpregnant rats that “sat quietly” on a treadmill within a metabolic box; this mean HR was 413, about 60 b/min higher than recorded by Dowell and Kauer (Dowell and Kauer 1993) in rats confined to their familiar cage.

This study design utilized a fixed (absolute) workload for the acute exercise stimulus. The increased body weight in the term pregnant animals could have resulted in an increased relative workload or decreased work efficiency on the treadmill compared to the nonpregnant rats. We are unaware of studies that have compared these variables in pregnant versus nonpregnant animals. It appears that in women, the absolute metabolic cost of weight-bearing exercise is higher in pregnancy; however, this increased submaximal VO_2_ is directly related to weight gain such that the metabolic cost/kg body weight is unaffected (Melzer et al. 2010). Pregnancy in women did not reduce work efficiency (the slope of VO_2_ to work rate) during treadmill exercise (Clapp 1989; Lotgering et al. 1991). As it appears that in women VO_2_max is generally unaffected during pregnancy (Melzer et al. 2010), the relative workload in late gestation as expressed as a % VO_2max_ (L/min) would be greater at a given absolute submaximal work rate. Applied to this study, the implication is that an enhanced sympathoadrenal response to the absolute workload would exaggerate the uterine artery vasoconstrictor response to exercise in the term pregnant rats. As we observed an attenuation of the exercise vasoconstrictor response in the term pregnant rats, we speculate that the observed difference in the uterine vasoconstrictor response between the nonpregnant and term pregnant rats may have been slightly underestimated.

### Summary

These data demonstrate that treadmill exercise triggers a reduction in uterine artery blood flow and vascular conductance in the rat, and the magnitude of these responses are blunted but not eliminated during late gestation. Qualitatively, a similar response occurs in pregnant rabbits. This implies that a pregnancy-related adaptation in uterine circulatory control occurs in rats as well as rabbits that limits the potential reduction in blood flow and thus oxygen delivery to fetuses during exercise performed during late gestation. Further work is necessary to elucidate the mechanisms underlying the alteration in uterine circulatory control.

## Conflict of Interest

No conflict of interest to report.
